# A detailed heterogeneous agent model for a single asset financial market with trading via an order book

**DOI:** 10.1371/journal.pone.0170766

**Published:** 2017-02-28

**Authors:** Roberto Mota Navarro, Hernán Larralde

**Affiliations:** Instituto de Ciencias Físicas, Cuernavaca, Morelos, México; East China University of Science and Technology, CHINA

## Abstract

We present an agent based model of a single asset financial market that is capable of replicating most of the non-trivial statistical properties observed in real financial markets, generically referred to as stylized facts. In our model agents employ strategies inspired on those used in real markets, and a realistic trade mechanism based on a double auction order book. We study the role of the distinct types of trader on the return statistics: specifically, correlation properties (or lack thereof), volatility clustering, heavy tails, and the degree to which the distribution can be described by a log-normal. Further, by introducing the practice of “profit taking”, our model is also capable of replicating the stylized fact related to an asymmetry in the distribution of losses and gains.

## Introduction

In the past five decades a great number of time series of prices of various financial markets have become available and have been subjected to analysis to characterize their statistical properties [1–5]. From the study of these time series, a set of statistical properties common to many different markets, time periods and instruments, have been identified. The universality of these properties is of interest because the size, the participants and the events that affect the changes of price (returns) in a certain market may differ enormously from those that affect another. Yet, these investigations show that the variations in prices indeed share non trivial statistical properties, generically called *stylized facts*. In this work we present and study a model of a financial market and its participants which reproduces these stylized facts.

The majority of approaches used today to model financial markets fall into one of two categories: statistical models adjusted to fit the history of past prices and Dynamic Stochastic General Equilibrium (DSGE) models. The first kind of models are able to produce reasonable representations and volatility forecasts of financial systems [6] as long as the statistical properties of the prices with which they were calibrated do not change by a large margin. The second kind of models assume a “representative agent” for each of the participant sectors in the financial system, each of these agents attempting to their utility [7]. To avoid creating deterministic dynamics without periods of depression or growth, DSGE models use exogenous stochastic terms which are supposed to mimic the varying conditions of the market, such as sudden peaks in the demand of a certain financial instrument or changes in the pricing of a commodity.

Despite of the fact that these models are capable of providing some explanations of the phenomena observed in financial markets, the premises over which they are built are crude approximations of reality [8, 9] and as a such they are not always useful to gain insight into statistical phenomena as rich at that observed in financial time series.

This situation has given rise to the exploration of financial systems as “complex systems” [10]. That is, to consider financial markets as something closer to what they actually are: systems where great number of different components interact amongst each other in a way that gives rise spontaneously to the observed macroscopic statistical properties.

Among the models which approach financial markets as complex systems, there is a particular kind called “Agent Based Models” which employ a bottom-up approach and allow the modeler to trace back the emergence of the macroscopic statistical properties of the system as a consequence of the microscopic behavioral traits of its constituent agents [11]. Several Agent Based Models have been created that are capable of reproducing stylized facts and provide possible microscopic explanations of their origins. These models have been constructed, in general, in one of two ways: models in which the agents do not use a particular set of strategies, but rather participate in the market in a random fashion, and models in which the agents follow different specific strategies inspired in actual strategies used by participants of real markets, as we do in this work. The first type of models usually make use of market trading structures similar to those used in real markets, such as double auction order books, and as a consequence, the price formation is directly driven by the offers (to buy and sell) supplied by the agents [12–21, 21, 22]. latter type of models usually have prices adjusted in a stochastic manner [23–26]. Thus, while models with “intelligent” agents employing different strategies in realistic market environments have been proposed before [27–32], our model is motivated by the behavior of market participants following the rules of thumb employed by real life traders, while keeping the model as simple as possible. In particular, we do not dwell on whether these rules of thumb have solid microeconomical foundations. Specifically, in our model, we consider two types of agent: technical and fundamental. Technical agents in our model follow a “Moving average oscilator” strategy [33], which is commonly used by real technical traders. These traders also incur in profit taking if the price of the asset exceeds a certain threshold. Heterogeneity among technical agents is achieved by assigning different parameters (“personalities”) to different subsets of the technical agent population. On the other hand, the fundamental agents in our model “choose” a fundamental price, and change it according to the influx of news as well as the distance to the positions of the rest of the agents in the market. The fundamental prices chosen by these agents, and their reaction to the incoming news, differ amongst agents, as happens in real life. Trading in the model is done through an order book.

Since the model is constructed trying to mimic behavioral patterns followed by the participants in real financial markets, we expect that, if these behaviors are succesfully captured, however simplified they may be, the resulting price statistics should reproduce the stylized facts observed empirically. Specifically, the stylized facts on which we focus in this paper, are the following:

Absence of auto-correlations: The auto-correlation function of the returns *R*(*t*) is essentially zero for any value of the lag (except at very short time in which there is a negative correlation “bounce” [2]). The absence of auto-correlations has been used as support for the efficient market hypothesis [34] since it implies that it is impossible to incur in arbitrage [35].

Volatility Clustering: Notwithstanding the absence of auto-correlations in the “raw returns” series, some non linear functions of returns do exhibit auto-correlations that remain positive for relatively long times. This behavior arises from the fact that the returns have a tendency to “agglomerate in time” in groups of similar magnitude but unpredictable sign [4].

Heavy tailed distribution of returns: The distributions of price changes in real financial time series do not have a normal distribution [4, 36, 37]. Instead, the distribution is characterized by having large positive values of the kurtosis (for instance, the kurtosis for the Standard & Poor’s index measured over time intervals of 5 minutes has been reported to have a value of *κ* ≈ 16 [38]). Further, studies of the complementary cumulative distribution of returns have shown that it behaves approximately as a power law with an exponent *β* ∈ [2, 4] [35, 36].

Asymmetry in the distribution of returns: In addition to being heavy tailed, it has observed that in many markets, large negative returns are more frequent than large positive returns. This asymmetry is behind the negative skewness in the returns distribution which has been reported in empirical studies [2].

Log-normal distribution of volatilities: The probability distribution of the volatility of individual firm shares and of indexes, defined as the average of the absolute returns over a time window, is well approximated by a log-normal distribution in its central part, while its tail is well adjusted by a power law with exponent *μ* ≈ 3 [39].

In the next section we present a detailed discussion of the agent based model we propose. The paper continues with a section in which we present the results obtained in simulations of the model and we focus on the stylized facts listed above, comparing the behavior of the model with representative empirical data. We also study the effect of varying the relative populations of agents as well as the parameters that control the practice of profit taking by the technical agents in the system. We end with a section of concluding remarks and perspectives.

## 1 Model

### 1.1 General aspects

The model represents a financial market in which *N* agents trade a single asset through a double auction order book in which the standing orders are registered until executed. In the model we only consider market and limit orders [40] of unit volume.

Like in actual financial markets, in the model, the population of agents is divided into two different sub-populations, with each sub-population employing one of two basic trading strategies: fundamental analysis -by which a “fundamental price” *p*_*f*_ is estimated, and then the traders attempt to take advantage of the deviations between *p*_*f*_ and its present trading price *P*_*t*_-; or technical analysis -by which the trader tries to identify and exploit trends in the price time series-.

These two types of strategies are representative of the main strategies used in real life trading and were first introduced in the Lux-Marchesi (LM) model [41]. The effects of these strategies on the dynamics of the price are opposed: while fundamental agents tend to stabilize the prices around the average value of their fundamental prices, technical agents tend to create periods of violent price changes.

The parameters controlling the behavior of each agent are assigned at the beginning of each simulation, and even if two agents belong to the same group (fundamental or technical) the difference in the values of their controlling parameters will generate different “personalities” within each strategy.

We make time run in discrete units corresponding to simulation steps and on each simulation step, each fundamental agent will engage in trading with a probability *p*_*active*_ while technical agents will be active when they observe a favorable trend or when they can obtain a high immediate profit, as will be explained later.

In our system, every agent is assigned unlimited credit, and, in contrast to [28], short selling is allowed. These two liberties are meant to ensure that an agent is able to engage in trading whenever it becomes active, thus providing the market with enough liquidity.

Although the model we propose includes the main components of the Lux-Marchesi [41] and the Chiarella C, Iori G and Perelló J [28] models, there are important differences in the way in which we designed both the agents and the market environment. Of central importance is the fact that in our model the process of price formation is directly governed by the demand and supply provided by the agents and, as in Chiarella C, Iori G and Perelló J [28], all the transactions are mediated through an order book. Another difference is the fact that by assigning different parameters, we include heterogeneity within each strategy. Further, in our model the only sources of “exogenous” randomness are, on one hand, the entry times of the fundamental agents; and on the other, the time of arrival and nature of the news in the system. The news elicit randomly distributed reactions from the fundamental agents, each of which estimates its own changes in the fundamental price and may adjust it if it differs too much from the prices at which other agents are bidding. This contrasts with Lux-Marchesi and Chiarella C, Iori G and Perelló J [28] in which a unique fundamental price performing a geometric random walk is assumed. Finally, while the details in the precise behaviors of our technical and fundamental agents differ from those of other models, we also include the possibility that agents can engage in profit taking, as happens in real markets.

### 1.2 Types of agents

#### 1.2.1 Technical agents

As mentioned above, technical agents employ “technical analysis” in an attempt to predict the future behavior of the price time series with the purpose of exploiting the knowledge of that future behavior.

**Fig 1:**
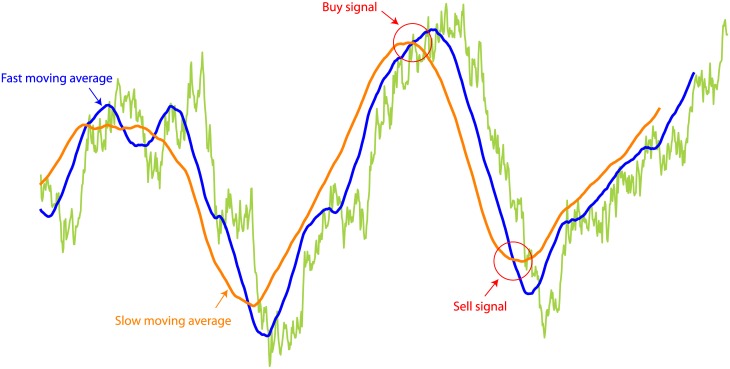
Moving average-oscillator (MAO). This is a common technical indicator which is formed by two moving averages of different window sizes that are constantly observed. The moving average with the largest window size is called *fast moving average* and the one with the smallest window is called *slow moving average*. When the fast moving average crosses the slow one from below, a signal to buy is generated; conversely, when the fast moving average crosses the slow from above, a signal to sell is generated.

In our model technical agents utilize a technique used in real life called Moving Average-Oscillator (MAO) [33], which consists of a pair of moving averages with different window sizes: a long period average called the *slow moving average*, and a short period average aptly called the *fast moving average*. The fast moving average is intended to capture the tendency of the price movements in a short term while the slow moving average has the purpose of capturing the long term trend. Fig 1 shows an example of this technical indicator.

When the fast average crosses the slow one from above, the MAO strategy suggests that this is a “signal to sell”, since the prices show a short term tendency to fall below the long term trend captured by the slow moving average. Similarly, a “signal to buy” occurs when the fast moving average crosses the slow one from below, since this can be interpreted as the prices having a short time tendency to rise above the long term trend.

We employ the MAO indicator in our model because while it is very simple and easy to implement, it is representative of the plethora of technical analysis tools and it is widely used in real markets [42].

In our model we use MAO indicators that differ in the window sizes of the two averages which compose them. For each of these indicators there is a population of technical agents following its evolution over time and engaging in trading as a result of the signals that the indicator generates. Further, when an indicator generates a signal to either a buy or sell, each technical agent following that particular indicator waits a particular time *t*_*wait*_ before entering the action suggested by the signal. This waiting time between the moment in which the signal is generated and the moment in which an agent enters its order is meant to allow the price time series to move in the direction predicted by the indicator. If the agents were to immediately enter their orders after they received a signal, they would not take advantage of the rise or fall in prices that the trends point to. The waiting time *t*_*wait*_ of each technical agent is drawn from a uniform distribution in the interval [0, *t*_*max*_], and assigned to each agent from the beginning of the simulation.

A consequence of the way in which the MAO indicator is constructed, is that the technical agents should have perfectly alternating order flows, with a sell order following a previously entered buy order and vice-versa. This alternation arises from the fact that the MAO indicator generates signals when the two moving averages cross each other and for any of the two directions of crossing: the fast average crossing the slow one from below or from above, the next direction will be necessarily of the opposite kind.

**Fig 2:**
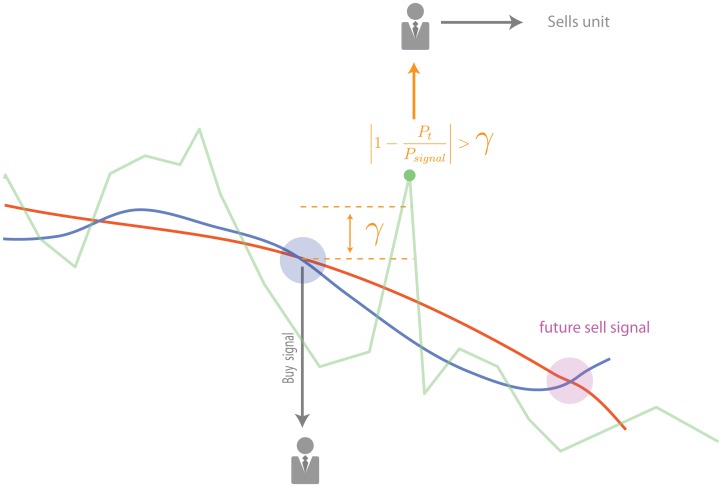
Profit taking mechanism. If after observing a signal to buy, the prices rise enough (in our case this is defined as the moment at which $\left|1-\frac{P_{t}}{P_{signal}}\right|$ exceeds a parameter *γ*), the technical agent will proceed to enter a sell market order. This practice is commonly used by traders to insure an immediate profit.

There is, however, another mechanism which compels a technical agent to engage in trading, aside from following the technical indicator. This mechanism is *profit taking* and it basically consists in selling the asset when the price is sufficiently high with respect to the price at which the last unit was bought, irrespective of whether the MAO indicator generates a sell signal or not, thus providing the agent with an immediate profit. This is implemented as follows, when a technical agent enters an order to the book while following the indicator, that agent registers the price at which the order was executed in a variable called *P*_*signal*_. If the price of the asset *P*_*t*_ deviates from *P*_*signal*_ by more than a factor *γ*, the agent will proceed to enter a new sell order; i.e. if after following a buy signal and entering the corresponding buy order to the order book the price of the asset is greater than (1 + *γ*)*P*_*signal*_, then the agent will place a sell market order, securing in this way an immediate profit. Fig 2 shows how profit taking is carried out in our model.

The profit taking mechanism is introduced in our model because it is a common practice in real financial markets and, as we will see, it turns out to have a strong effect on the return statistics. Finally, technical agents in our model act almost immediately after receiving a signal from their technical indicator and decide whether to buy o sell only regarding the present price of the asset. Thus, only market orders will be issued by this type of agent, any consideration of the value of the asset used for establishing a target price at which to enter limit orders is left to the fundamental agents.

#### 1.2.2 Fundamental agents

A fundamental analysis trading strategy is based on two basic premises: the first one being that every asset has an intrinsic “fundamental price” *p*_*f*_, and the second one, that in the short run, this fundamental price may be incorrectly estimated by the market participants but that in the long run, the market will correctly value the asset and its price will eventually reach the fundamental price *p*_*f*_. An agent following a strategy of this kind will therefore buy an asset when the price at which it is being traded is below his estimation of its fundamental price *p*_*f*_ and will sell the asset when its price is above *p*_*f*_. In this way a person following a fundamental strategy will take advantage of the differences between the prices at which the asset is traded over time and the fundamental price; until the asset finally reaches said fundamental price.

When a fundamental agent becomes active, there are three available actions that this agent can engage in: either to buy a unit of the asset, to sell it (even short sell) or to abstain from either. The decision of whether to buy, sell or abstain from participating will depend on the position of the agent’s fundamental price *p*_*f*_ relative to the price of the nearest best order (best ask or best bid).

If *p*_*f*_ > *B*_*sell*_, where *B*_*sell*_ is the price of the best ask, the agent will proceed to buy since there are agents willing to sell for less than what the agent considers to be the correct price. Similarly if *p*_*f*_ < *B*_*buy*_, where *B*_*buy*_ is the price of the best buy, the agent will proceed to sell since there are agents willing to buy offering more than the correct price. If neither of these two conditions is fulfilled, i.e. if *B*_*sell*_ > *p*_*f*_ > *B*_*buy*_ then there will be no competitive offers, since the lowest price at which the agent could buy a unit of the asset is higher than *p*_*f*_, and the highest price at which it could sell a unit is lower than *p*_*f*_. Thus, when this condition arises the agent will abstain from participating in the market.

**Fig 3:**
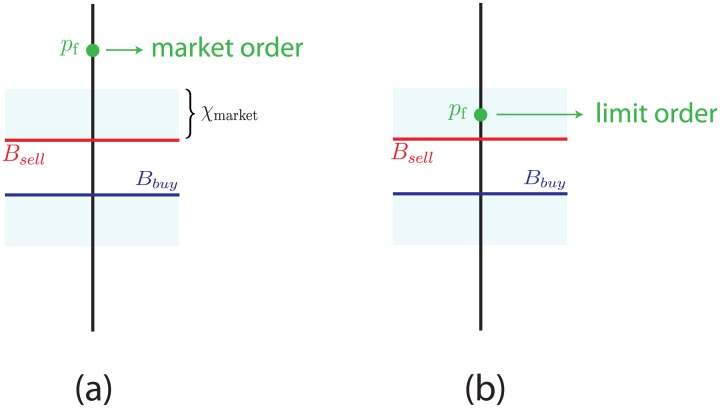
Order selection algorithm for fundamental agents. In (a) we show the conditions that lead to a fundamental agent to introduce a market order: if the fundamental price *p*_*f*_ is higher by more than a threshold *χ*_*market*_ (specific to each trader) with respect to the price of the nearest best order, the agent will proceed to enter a market order. Otherwise, the agent will proceed to enter a limit order (b). In the figure the orders would be “buy” orders as the agent’s fundamental price lies above the best ask.

When an agent decides to buy or sell, the decision to do so by entering a limit or a market order will depend on the distance between *p*_*f*_ and the price of the nearest best order. Specifically, if the agent decides to buy, it will do so by emitting a market buy order when its fundamental price is above the price of the best sell offer by more than a certain threshold *χ*_*market*_, i.e. when *p*_*f*_ > *B*_*sell*_(1 + *χ*_*market*_), and it will emit a limit buy order when *p*_*f*_ is below this threshold. Similarly, when the agent decides to sell, it will do so by emitting a market sell order if its *p*_*f*_ is below the best buy offer by more than the threshold *χ*_*market*_, i.e. when *p*_*f*_ < *B*_*sell*_(1 − *χ*_*market*_), otherwise it will emit a limit sell order. Just like every other parameter defining the behavior of a fundamental agents, every agent is assigned an individual threshold *χ*_*market*_ from the beginning. Fig 3 shows this decision making algorithm.

On the occasions in which a fundamental agent decides to enter a limit order, the actual price of the order is extracted from a shifted symmetric exponential distribution of the form:

$$\begin{array}{c} f\left(x;\lambda_{limit},\mu_{spread}\right)=\lambda_{limit}e^{-|\lambda_{limit}\left(x-\mu_{spread}\right)|} \end{array}$$

where *μ*_*spread*_ is the average price of the best orders: $\mu_{spread}=\frac{1}{2}\left(B_{sell}+B_{buy}\right)$. By assigning the prices of limit orders in this way, they will have a greater tendency to cluster around *μ*_*spread*_ which is a representative measure of the central price at which the market participants are valuing the asset. This behavior is intended to reflect the situation in which the prices are not good enough to enter a market order, so the fundamental agents will proceed to bargain with limit orders at prices that will be close to the central price in the market.

**Fig 4:**
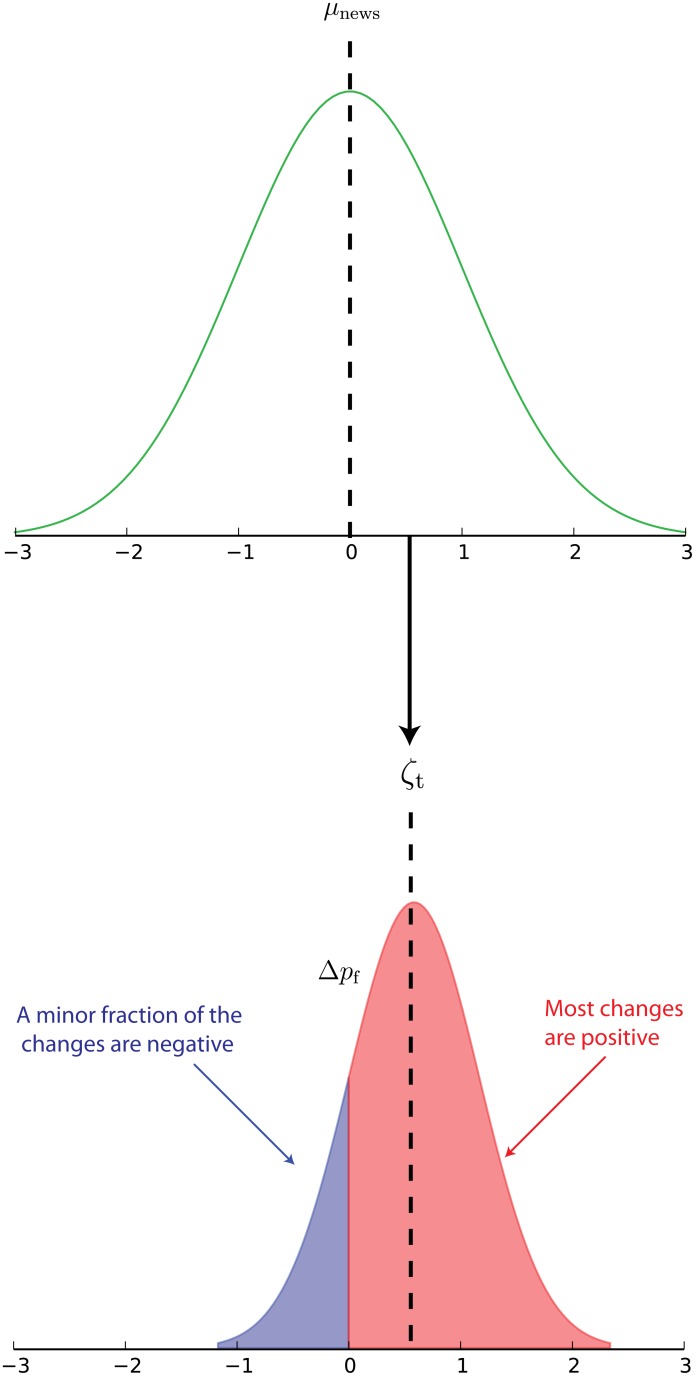
News and their effects on fundamental prices. We model news as a sequence of IID Gaussian random variables. When a realization of this sequence, representing news being issued, occurs, the fundamental prices of each agent are adjusted from *p*_*f*_ to *p*_*f*_ + Δ_*p*_*f*__ with Δ_*p*_*f*__ extracted from another normal distribution whose mean is equal to the value of the current news. In this way when highly positive news arrive, the majority of fundamental price changes will be positive; conversely, when highly negative news arrive, most price changes will also be negative.

In real life, *p*_*f*_ is determined by each fundamental trader, and then adjusted as time goes by, according to the appearance of news concerning the well being of whatever underlies the asset. To include this feature of fundamental analysis in our model, we introduce a flow of news modeled as a sequence of IID random variables *ζ*_*t*_ taken from a normal distribution with mean *μ*_*news*_ and variance *σ*_*news*_. The time intervals betwen succesive news are taken from a Poisson distribution. Here, *ζ*_*t*_ represents the mean value by which the news will change the fundamental prices of the asset. When, in the context of our model, news are issued at a given time *t*, each fundamental agent adjusts its fundamental price from *p*_*f*_(*t*) to *p*_*f*_(*t*) + Δ*p*_*f*_(*t*) where Δ*p*_*f*_(*t*) is again extracted from a normal distribution with mean *ζ*_*t*_ and variance *σ*_Δ*p*_*f*__ as illustrated in Fig 4. Thus, the majority of fundamental agents will change their prices accordingly with the sign of *ζ*_*t*_, however, depending on the magnitude of the news, some agents may even extract a Δ*p*_*f*_ with an opposite sign to *ζ*_*t*_. This diversity of response to a news item attempts to reflect the possibility of diverse interpretations of the information by the fundamental agents. The fundamental price of each agent is chosen from a uniform distribution at the beginning of a simulation.

Finally, although a fundamental agent bases its trading strategy in the differences between its fundamental price and the prices at which the market values the asset, if too large a difference is present, the agent will try to get closer to the central market price *μ*_*spread*_. This feature is meant to capture the attention that a fundamental agent pays to the opinions of the whole population of agents, which constitutes a mild manner of “herding behavior”. If the valuation of the fundamental price that an agent has is too far from the price at which it is being traded, the agent will move its fundamental price closer to the central price *μ*_*spread*_. This can be interpreted as a precautionary move by the agent since such a big difference between *p*_*f*_ and *μ*_*spread*_ could point to information that was not incorporated in the determination of his fundamental price, or that an ineffective incorporation of the available information was made.

To determine when the difference between *p*_*f*_ and *μ*_*spread*_ is “too big”, each agent compares this difference with a threshold *χ*_*opinion*_, if at the time a fundamental agent becomes active, such agent observes that

$$\begin{array}{c} \chi_{opinion}<\left|1-\frac{p_{f}}{\mu_{spread}}\right| \end{array}$$

Then the agent will adjust its price to get closer to *μ*_*spread*_ in the following way:

$$\begin{array}{c} p_{f}=\{\begin{array}{cc} \mu_{spread}(1+\chi_{opinion}), & if\;\;p_{f}\geq \mu_{spread}\\ \mu_{spread}\left(1-\chi_{opinion}\right), & if\;\;p_{f}<\mu_{spread} \end{array} \end{array}$$

Thus, the agent will get as close to *μ*_*spread*_ as the maximum tolerance (*χ*_*opinion*_) between its opinion and the opinion of the population (*μ*_*spread*_) allows.

## 2 Results

In this section we present the results obtained in various simulations. Although these results correspond to a particular set of values for the parameters, reasonable changes in the values of these parameters generate the same qualitative properties in the statistics of the model. It is of critical importance for the stability of the system to have a flow of limit orders (liquidity) capable of filling the gaps that are created when market orders enter the order book. To achieve this, the parameters that govern the flow of limit and market orders emitted by the agents must not give rise to bursts of market orders with a volume so large that one side of the order book is emptied. It is in this sense that we speak above of reasonable changes in the values of the parameters. Thus, for example, if we were to allow greater volumes of market orders to be placed within shorter time windows, say, by including a larger number of technical agents in a simulation, then, the parameters that affect the input of limit orders must be chosen accordingly, in such a way that the fundamental agents have enough time to restore the liquidity consumed by the increased number of market orders. Thus, we calibrated the model to achieve stability in the simulations and to reproduce the statistical properties of the returns observed in real life and did not consider calibrating the model to reproduce the order book stylized facts [36] Chiarella C, Iori G and Perelló J did in their work [28].

Unless otherwise stated, the following results were obtained with a population of 1000 fundamental agents and 1500 technical agents divided into two groups of 750 agents with technical indicators made of moving averages with window sizes of 4000 and 2000 time steps for one group and 2000 and 1000 time steps for the other. The other parameter values used for this run are shown in Table 1.

**Table 1:** Values of the parameters corresponding to the results presented in this paper (ranges indicate that the parameters for each agent were taken from a uniform distribution in within the specified values).

Parameter	Value
*P*_*active*_	0.15
*p*_*f*_(initial)	[20.0, 25.0]
*χ*_*market*_	[0.005, 0.25]
*χ*_*opinion*_	[0.01, 0.1]
*σ*_Δ_*p*_*f*___	0.2
*λ*_*limit*_	3
*μ*_*news*_	0
*σ*_*news*_	0.1
*f*_*news*_	100
*γ*	0.01
*t*_*wait*_	[0, 50]

As is frequently the case for many financial models, some of the parameters defined in our model may not have a clear connection to observables in real life, and even when observables similar to the parameters in our model exist, attempting to estimate their values is somewhat ambiguous. Thus, we chose values which allowed the simulations to run in a stable manner and that generated statistical properties similar to those observed in real markets. Interestingly, the model is rather robust and produces similar relevant results for a wide range of parameter values. The values of the parameters we employed for the results we present below are therefore, just an election among many different elections we made within the range of useful parameter values.

**Fig 5:**
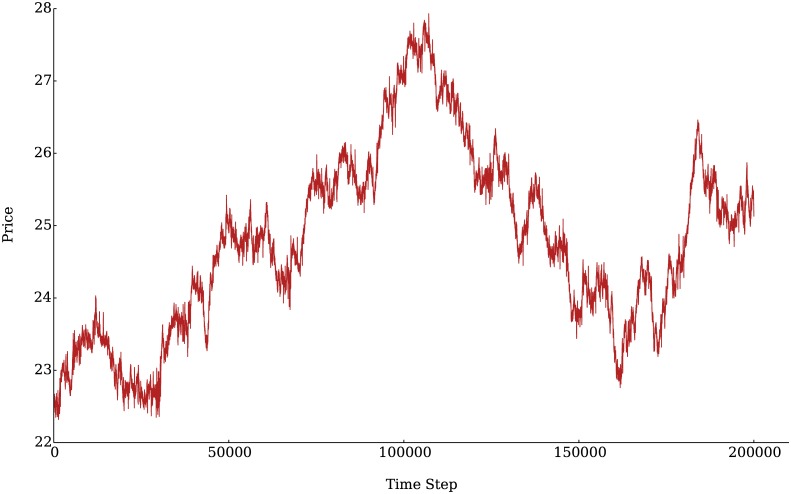
Representative time series of asset prices, determined as the last price the asset was traded at each time step (“closing price”).

**Fig 6:**
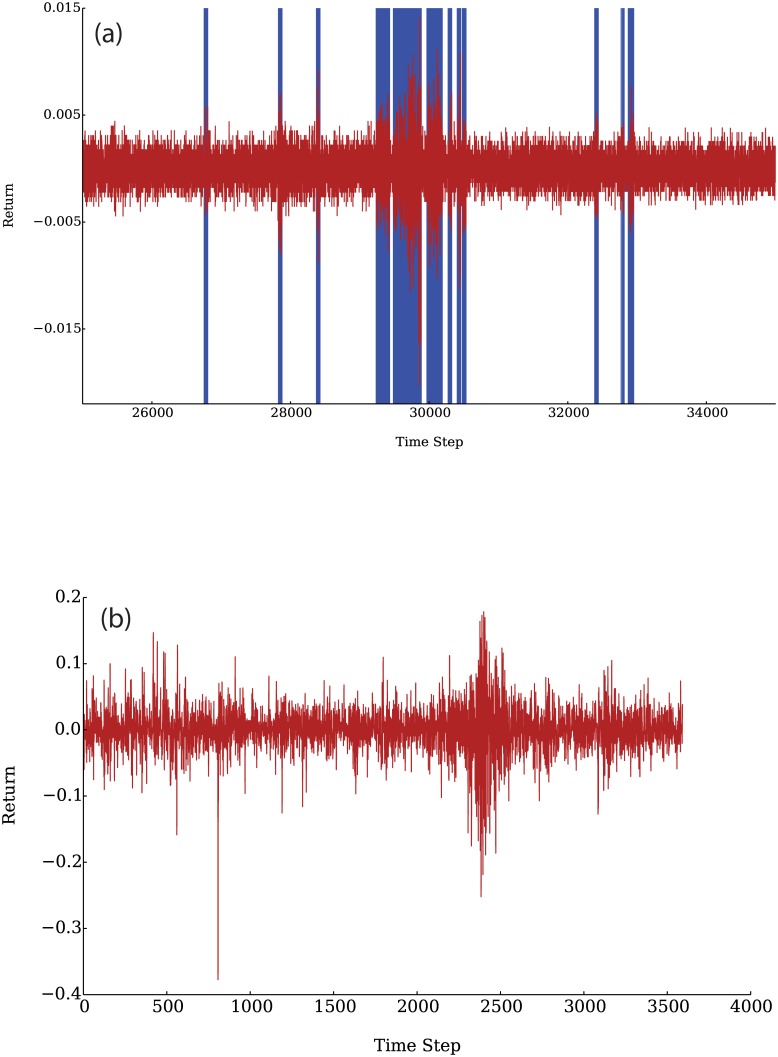
Returns time series for the simulation with a time lag *τ* = 1 (a) and comparison with empirical data from Consol Energy Inc (b). The blue shaded regions show the times in which technical agents were active, as can be seen, these times coincide with the periods with the largest changes of price. Data obtained from QuantQuote [43].

We begin by showing the time series corresponding to the prices and logarithmic returns, defined as *r*(*t*) = log(*P*_*t*_/*P*_*t*−*τ*_), for a given lag *τ*, generated by our model. These are shown in Figs 5 and 6(a) respectively. The blue bars in Fig 6(a) signal the time steps in which technical agents were active. The bursts of greater volatility coincide with the activity of the technical agents while the times in which only fundamental agents were active (trading) present lower volatility.

**Fig 7:**
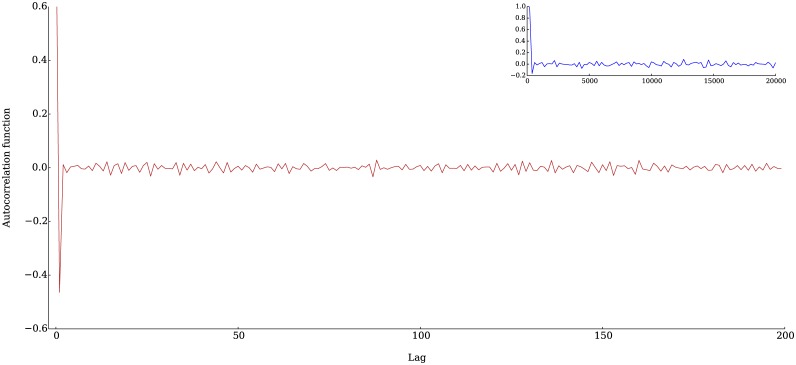
Auto-correlation functions of returns. There are essentially no correlations for any value of the lag, except for a negative correlation that lasts for a few steps at the beginning. This phenomenon is also observed in real returns series and has been called *bid-ask bounce* [2]. The main figure corresponds to the autocorrelation function of the returns calculated every time step and the inset figure to the returns calculated every 50 steps.

In Fig 7 we show the auto-correlation function of the returns, the blue line corresponds to the returns calculated time step by time step. In the inset we show the auto-correlation function for returns calculated every 50 steps, in both cases it can be seen that the auto-correlation is essentially zero for any value of the lag. It is interesting to note that the phenomenon know as “bid-ask bounce” can be observed in the returns generated by our simulations. This phenomenon consists in the presence of negative values of the auto-correlation function at very short lags and it is attributed to the fact that most transactions take place near the best ask or best bid and tend to bounce between these two values [2].

**Fig 8:**
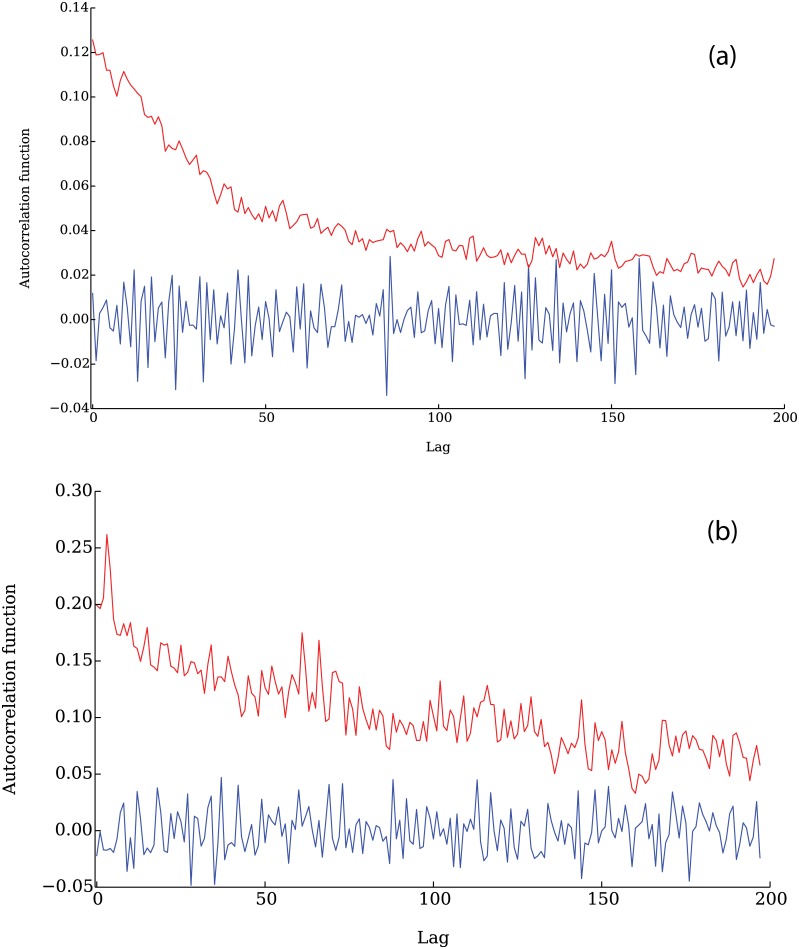
Returns auto-correlation function for the simulation (a) and comparison with empirical data from Airgas Inc (b). While the auto-correlation of the direct returns (blue lines) is zero, the auto-correlation of the absolute value of the returns (red lines) remains positive for a long period of time, and decays slowly to zero. Data obtained from QuantQuote [43].

In Fig 8(a) we present the comparison between the auto-correlation of the returns (blue line) and the auto-correlation of the absolute value of the returns (red line). We observe that the auto-correlation function of the absolute returns remains positive over a long time interval, and that it decays slowly to zero. Fig 8(b) illustrates the same auto-correlation functions for a representative company listed in the Standard & Poor’s 500.

**Fig 9:**
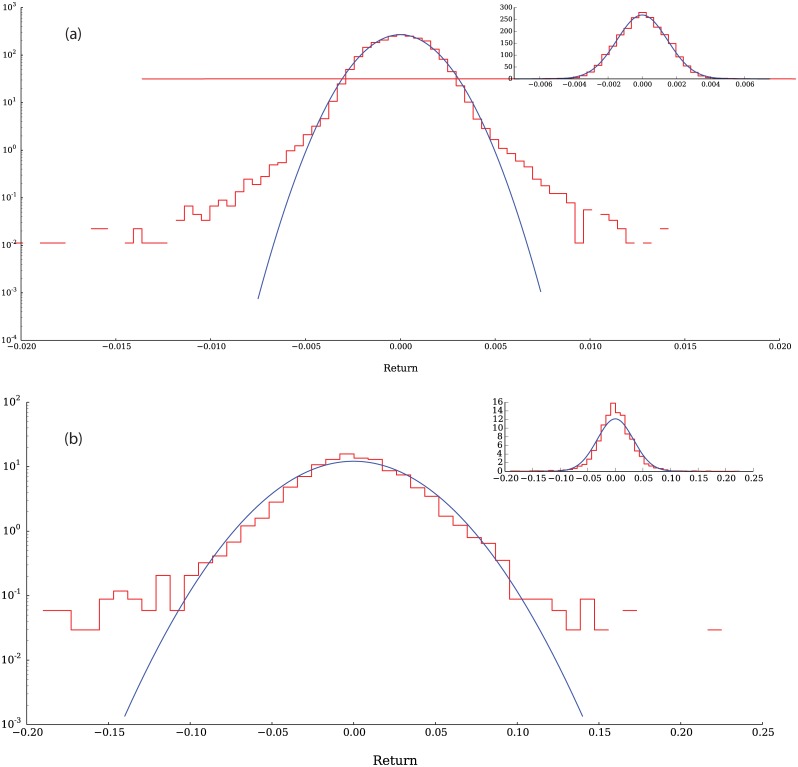
Returns PDF from the simulation (a) and comparison with empirical data from United States Steel Corporation, Inc. The tails of the distribution (red line) are clearly heavier than those of a normal distribution (blue line). Data obtained from QuantQuote [43].

Fig 9(a) shows the distribution function of returns from our model. This distribution shows heavier tails than a normal distribution with the same mean and standard deviation and it is possible to observe that the left tail is heavier than the right one. For comparison, Fig 9(b) illustrates the distribution function of returns for a representative company listed in the Standard & Poor’s 500.

**Fig 10:**
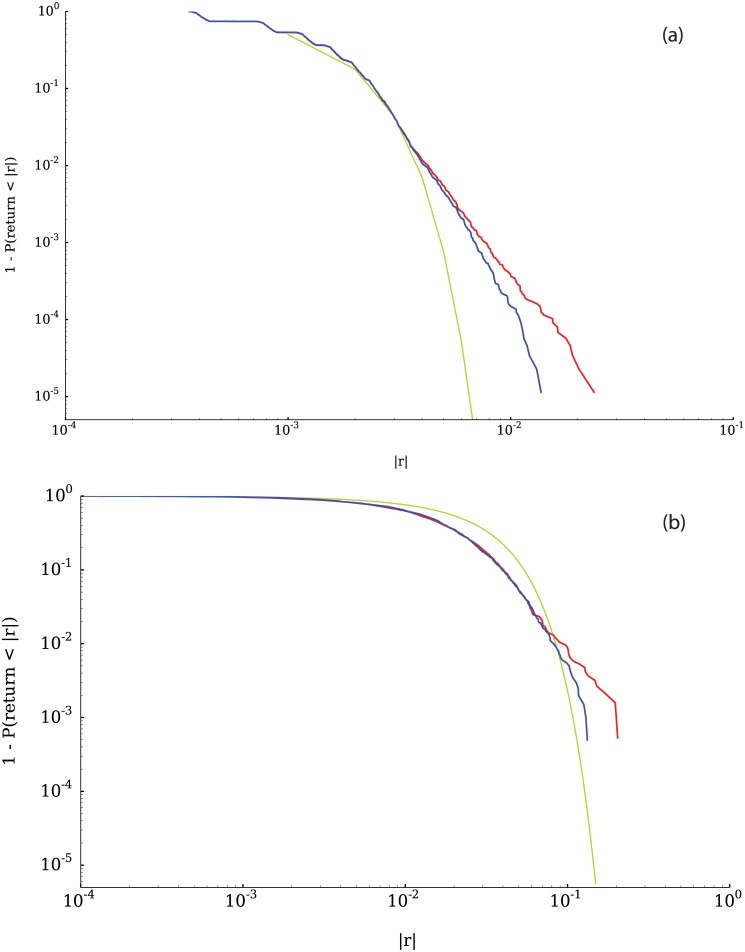
Comparison of the positive and negative returns CDF from the simulation (a) and from empirical data for Anadarko Petroleum Corp (b). It can be seen that the left tail of the distribution (red line), corresponding to the negative returns, is heavier than the right tail (blue line), corresponding to the positive returns. This is related to the negative skewness observed in the distribution.

**Fig 11:**
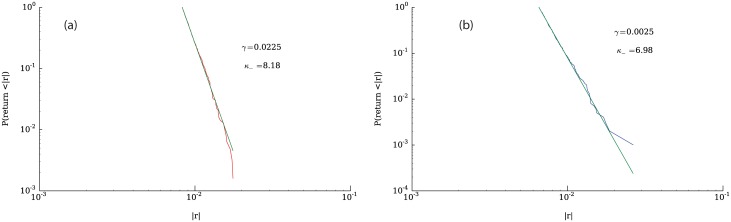
Complementary cumulative distribution functions and their powerlaw fits for *γ* = 0.0025. Here *κ*_−_ and *κ*_+_ are the exponents of the powerlaw fits for the left and right tail correspondingly. Fig (a) shows the negative returns and Fig (b) the positive returns.

**Fig 12:**
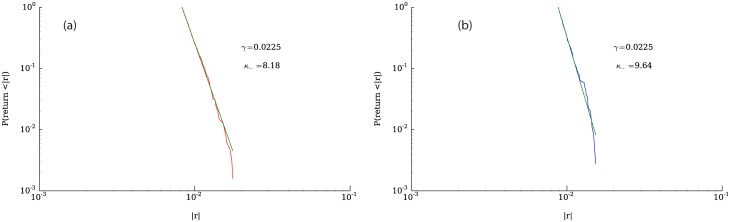
Complementary cumulative distribution functions and their powerlaw fits for *γ* = 0.0225. Fig (a) shows the negative returns and Fig (b) the positive returns.

**Fig 13:**
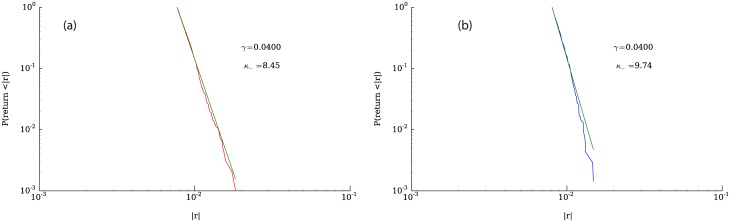
Complementary cumulative distribution functions and their powerlaw fits for *γ* = 0.0400. Fig (a) shows the negative returns and Fig (b) the positive returns.

**Fig 14:**
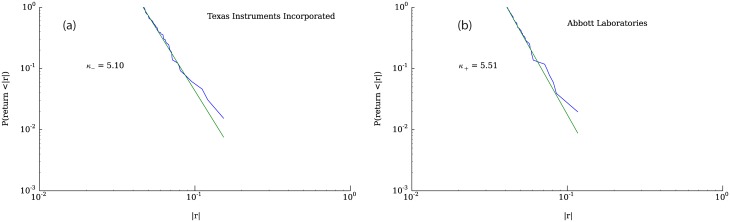
Powerlaw fits of two companies (Abbott Laboratories and Texas Instruments) with tail indexes greater than 3. Data obtained from QuantQuote [43]. Fig (a) shows the negative returns and Fig (b) the positive returns.

Fig 10(a) shows the cumulative complementary distribution of positive and negative returns, highlighting the asymmetry between losses and gains. The tail of the distribution of negative price changes is significantly heavier than the distribution of positive changes, a fact that is consistent with the negative skewness displayed by the returns distribution. Fig 10(b) illustrates the corresponding distributions for a representative company listed in the Standard & Poor’s 500. In addition to the asymmetry, it can be seen in Fig 10(b) that the tails of the distribution of returns seem to follow power law behavior. To test how well a power law fits the data, we used the python package “powerlaw” [44]. Figs 11(a) and 11(b), 12(a) and 12(b) and 13(a) and 13(b) show fits for three different values of the parameter *γ*. As can be seen in the figures, both tails of the distribution are rather well described by power laws, although the values for the exponents are not around 3, which is the average tail index reported in [45] and [37] (however see [46]. Nevertheless, some of the values we obtain correspond to those measured in the returns time series of some companies, examples of which can be seen in Fig 14(a) and 14(b).

The goodness of fit tests performed by the “powerlaw” package throw as a result the log-likelihood ratio *R* between two different candidate distributions. In this test *R* > 0 (respectively *R* < 0) when the first distribution is more (less) likely to describe the data than the second distribution [47]. To assess how much the sign of *R* was affected by the statistical fluctuations, the significance *p*, gives the probability of measuring a given value of *R* under the assumption that its real value is close to zero. A small value of *p* means that it is unlikely that the measured value of *R* is a product of the fluctuations, and, as a consequence, that its sign can be trusted as an indicator of which distribution provides a better fit for the data. The average values of *R* and *p* for simulations with different values of *γ* are presented in Table 2. For each value of *γ* in the table an ensemble of 50 simulations was run and the mean values of the loglikelihood for the left tail (<*R*_−_>) and right tail (<*R*_+_>) as well as the significance values <*p*_−_> and <*p*_+_> are presented.

**Table 2:** Values of the mean log-likelihood ratios <*R*> between the powerlaw and lognormal fits and of the mean significance values <*p*>. The values are presented for three representative cases of our model with different values of *γ*. Here <*R*_−_> and <*R*_+_> stand for the log-likelihoods of the left and right tails, correspondingly. Similarly, <*p*_−_> and <*p*_+_> stand for the mean significance values for the left and right tails.

	<*R*_−_>	<*p*_−_>	<*R*_+_>	<*p*_+_>
*γ* = 0.0025	−0.006	0.595	−0.032	0.057
*γ* = 0.0225	0.004	0.597	−0.053	0.623
*γ* = 0.0400	−0.050	0.609	0.203	0.489

Table 3 shows the mean loglikelihood ratios and significance values measured in the empirical data. From the empirical data set it can be seen that although the ratios point to a power law as the best fit when compared to a lognormal distribution, the significance values are again high enough (>0.10) to make inconclusive the test. Similarly, in the data set generated from the simulations, the significance values are too high to ascertain whether a powerlaw distribution is a better fit than a lognormal. Nevertheless, the power law fits seem to be a very good description of the behavior in both tails of the distribution for all three cases of *γ*, which span the range from very frequent to very scarce engagement in profit taking.

**Table 3:** Values of the mean log-likelihood ratios <*R*> between the powerlaw and lognormal fits and of the mean significance values <*p*> for the empirical data.

<*R*_−_>	<*p*_−_>	<*R*_+_>	<*p*_+_>
0.258	0.399	0.403	0.338

**Fig 15:**
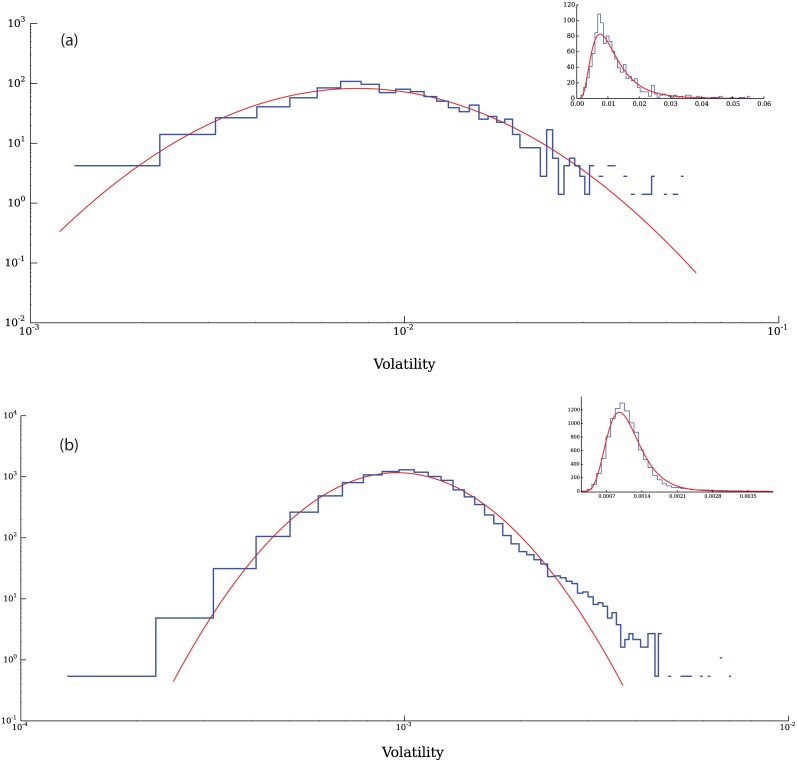
Distribution of volatilities for a simulation with both fundamental agents and technical agents (a) and comparison with empirical data from the Standard & Poor’s 500 (b). It can be seen in (a) that while the distribution of returns is not well described by a log-normal distribution, the central region is qualitatively similar to one, but the right tail is considerably heavier. Data obtained from Yahoo Finance.

In Fig 15(a) we present the distribution of volatilities measured as the average of the absolute value of returns |*r*(*t*)| over a time window *T* = *n*Δ*t*, i.e.

$$\begin{array}{c} V_{T}\left(t\right)=\frac{1}{n}\sum_{t^{{}^{\prime }}=t}^{t+n-1}|r(t^{{}^{\prime }})| \end{array}$$

**Fig 16:**
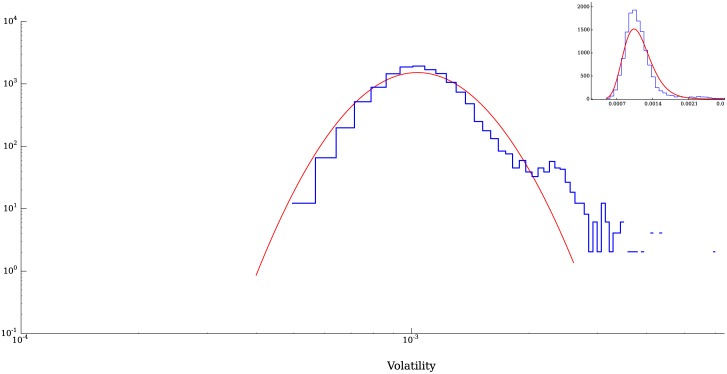
Volatilities of a simulation without technical agents. When only fundamental agents are used in a simulation, a log-normal distribution is a remarkably good description of the distribution of volatilities.

For the present result we took values of *n* = 30 and Δ*t* = 1 time steps. The distribution of volatilities is not well described by a log-normal distribution, however, the central part of the distribution may be approximated by one [39]. On the other hand, when we remove the technical agents from the simulation, the volatilities are remarkably well described by a log-normal distribution as shown in Fig 16, which corresponds to a run with the same parameter values described in Table 1 without technical agents.

To assess how well a lognormal distribution fits the volatilities, we performed a Kolmogorov-Smirnov test on the empirical data and on four different sets of data generated with our model. The p-values obtained from these tests are presented in Table 4. Even in the case with *γ* = 0.0025 which generated data which clearly deviates from a lognormal distribution at the tails, the average p-value is still high enough to make the rejection of the lognormal hypothesis difficult. The values obtained with the model are very similar to the value of the average p-value measured from the empirical data, which is at 0.47.

**Table 4:** Values of the mean p values corresponding to the goodness of fit of a lognormal distribution for the distribution of volatilities for four representative cases of our model with different values of *γ*.

	*γ* = 0.0025	*γ* = 0.0150	*γ* = 0.0300	*γ* = 0.0400
p-value	0.21	0.42	0.49	0.50

**Fig 17:**
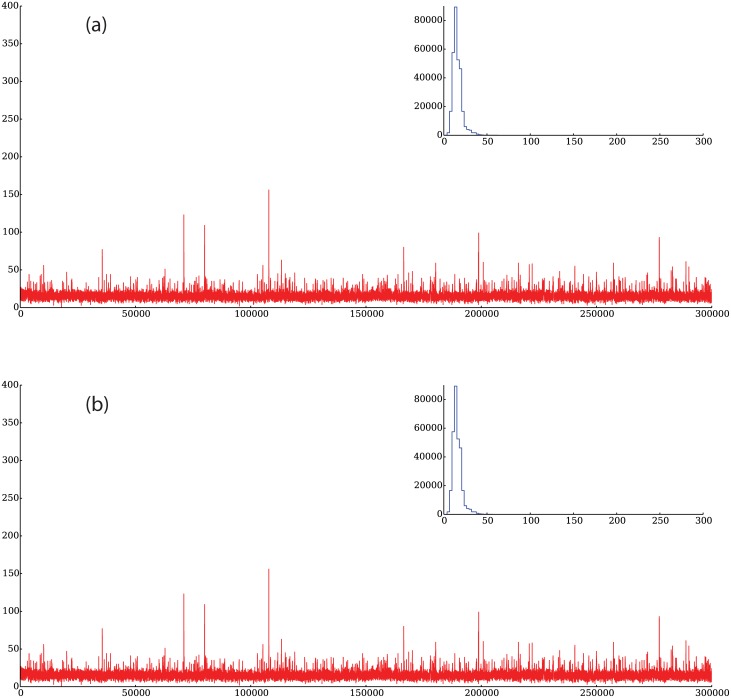
Representative trading volumes for runs of the model without technical agents (a) and with technical agents (b). As it can be seen, there are large fluctuations of the volume when technical agents are included in a simulation (b) but when only fundamental agents are present (a), the volume forms a steady flow with little deviations from its mean. The insets in each figure show the distribution of flows.

This similarity in the central part of the volatility distributions in the scenarios with and without technical agents, along with a similar result obtained by Schmitt T, Schäfer R, Münnix M and Guhr T [16] with their model, in which the agents place orders with exponentially distributed volumes, is of interest since the flows of orders are very different in both cases (see Fig 17(a) and 17(b)), yet, the majority of the volatilities can be described by log-normal distributions. This result suggests that the order book mitigates in some sense, the variations in the shape of the incoming order “signal”, in such a way that the variations in price (the volatilities) are not strongly affected by changes in the distribution of orders placed into the book.

**Fig 18:**
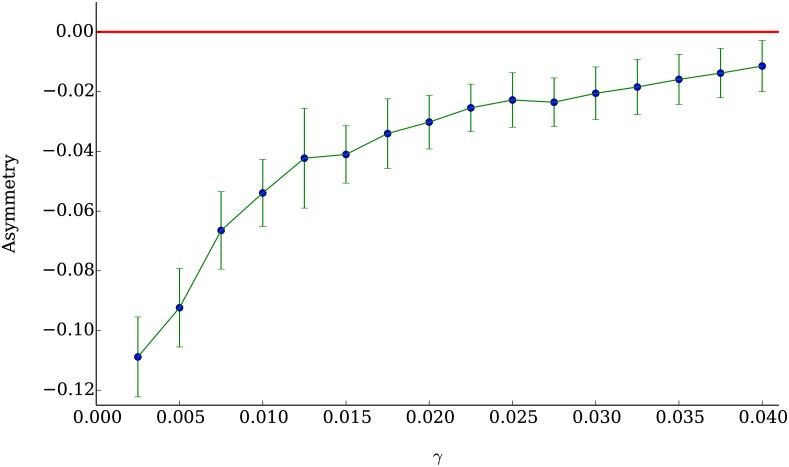
Mean skewness of the distributions of returns as a function of the profit taking threshold *γ*. As *γ* becomes smaller, more profit taking takes place and the mean skewness of the distributions of returns becomes more negative.

In Fig 18 we plot the values of the average skewness of an ensemble of 50 simulations (for every point in the plot) as a function of the parameter *γ*. As explained above, this parameter controls how often the population of technical agents engage in profit taking. In the framework of our model, this behavior is the cause of the asymmetry between losses and gains in the distribution of returns, since by enanging in profit taking, the population of technical agents creates large falls in the price of the asset.

The mean skewness we measured in the empirical data obtained from QuantQuote [43] has a value of −0.33; close to the minimum average skewness obtained in our model with the technical agents population engaging frequently in profit taking at *γ* = 0.0025. The number of companies with a skewness within the interval [−0.5, 0] is 199, which represents 39.8% of the companies listed in the S&P500.

**Fig 19:**
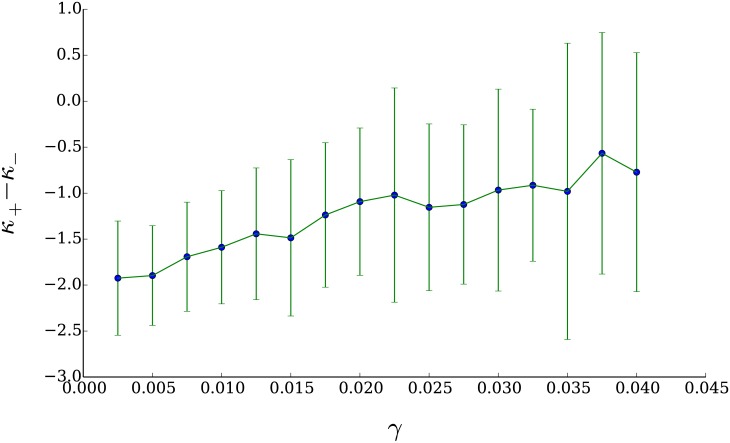
Mean difference of the exponents *κ*_−_ and *κ*_+_ of the power law fits for the absolute value of the negative (red) tail and the positive (blue) tail of the distributions of returns. This difference tends to decrease as *γ* becomes larger, suggesting that the tails of the distribution tend to collapse one on top of the other as the technical agents engage less frequently in profit taking.

In Fig 19 we present another test that relates the asymmetry of the distribution of returns to the practice of profit taking. In this figure we present the differences between the exponents of the power law fits for both the positive tail and the absolute value of the negative tail of the distribution of returns for several ensembles of 50 simulations in which we varied the parameter *γ*.

**Fig 20:**
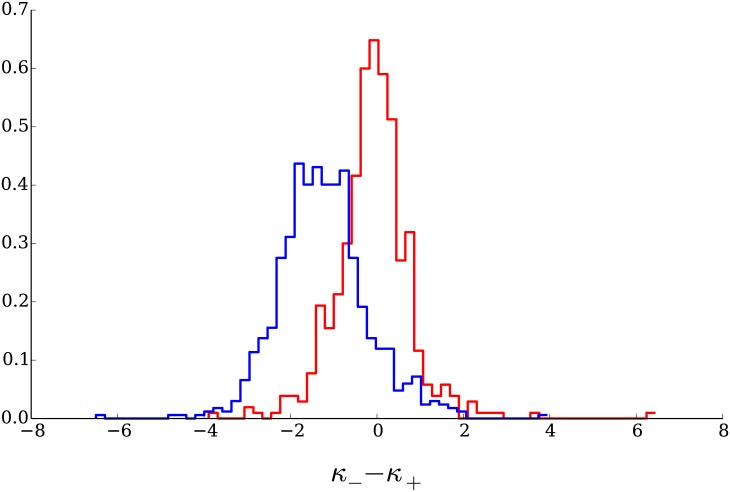
Distribution of the differences between the negative and positive tail exponents of the powerlaw fit. The red histogram corresponds to the values of the empirical data, the blue one corresponds to the data generated by the model. A significant number of companies present values in the range generated by our simulations.

As can be seen in Fig 19, we obtain mean values of the difference *κ*_−_ − *κ*_+_ in a range of [−1.92, −0.56]; the distribution of values for this difference as measured in the empirical data is in the Fig 20. The differences between the exponents for the power law fit obtained from the data generated with our model present a significant overlap with the empirical ones.

**Fig 21:**
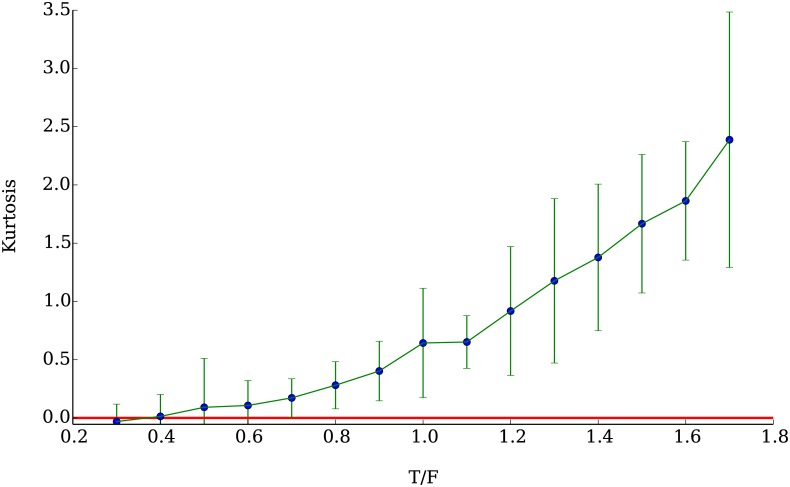
Mean kurtosis of the distributions of returns as a function of the ratio of technical to fundamental agents. As the proportion of technical agents increases, so increases the mean kurtosis in the ensemble of simulations.

Similarly, in Fig 21 we plot the average kurtosis of an ensemble of 50 simulations as a function of the fraction of technical agents in the population, in analogy to what is done in [48]. The kurtosis shows an increase with the number of technical agents, which strongly suggests that they are responsible for the deviations from normal behavior observed in the distribution of returns. Empirically, the kurtosis measured on the various companies listed in the S&P500 span a wide range of values, with some companies having a kurtosis higher than 100. With our model, we were able to produce kurtosises as high as 7 when the population of technical agents was almost twice the size of the population of fundamental agents. Unfortunately, using higher ratios without compromising the stability of the simulations requires a much larger total population of agents which is beyond our computational capacities.

## Conclusion

In this work we studied an agent based model of a single asset financial market with agents employing simple heuristic rules, which is capable of replicating the stylized facts reported in the literature. As in the LM model [41], we divided the population of agents into two groups according to the type of trading strategy they use: fundamental agents and technical agents. Further, we added heterogeneity within each group by varying the values of the parameters that control each agent’s behavior. Our aim was to create a model whose agents behaved realistically, as in the LM model, but with equally realistic market structures, namely, trading via a limit order book. We find, in accordance with previous models, that when the population of agents include technical agents, the returns present volatility clustering and a heavy tailed distribution. Further, we found that essentially no autocorrelation of the returns was present for any configuration of the populations. In addition to these main stylized facts, we find that when we allow the population of technical agents to engage in profit taking, the distribution of returns displays negative skewness and an asymmetry between losses and gains appears. By varying the frequency with which technical agents engage in profit taking, we can generate return distributions with varying degrees of separation in the tails. This dependence of the skewness over the frequency of profit taking suggests that this practice may be one of the causes of the appearance of the asymmetry in real financial markets.

Regarding the distribution of volatilities we find that only its central part is qualitatively similar to a lognormal distribution when technical agents are included in the population. If, on the other hand, we only include fundamental agents, the volatilities are remarkably well described by a lognormal distribution. The similarity of the volatility distributions in both scenarios, at least in the central part, suggests that its shape may not be strongly dependent on the detailed properties of the flow of incoming orders, since this flow varies significantly when technical agents are inserted in the population as compared with a population comprised entirely of fundamental agents.

We accompany our results with empirical data from real financial series chosen to illustrate the various stylized facts reproduced by our model.

In its present state, the model represents a single asset market, however, it is simple enough to be extended in several ways. For instance, an interesting extension to the model would be to increase the number of assets in the market and to limit the credit available to each agent. By doing this, the well being of the different “companies” associated to the different assets could become correlated depending on the shifts of the demand for each asset. Thus, we could inquire into the nature of these correlations, and how they are related to the composition of the population of agents. Another interesting modification would be the introduction of sequences of catastrophic news. The model will allow us to study how fast and in which way the market recovers to states observed previous to the arrival of the catastrophic news, if it recovers at all, and if the composition of the population affects this recovery.
